# Oldest Pathology in a Tetrapod Bone Illuminates the Origin of Terrestrial Vertebrates

**DOI:** 10.1371/journal.pone.0125723

**Published:** 2015-05-04

**Authors:** Peter J. Bishop, Christopher W. Walmsley, Matthew J. Phillips, Michelle R. Quayle, Catherine A. Boisvert, Colin R. McHenry

**Affiliations:** 1 Ancient Environments Program, Queensland Museum, 122 Gerler Rd, Hendra, Queensland, 4011, Australia; 2 School of Earth, Environmental and Biological Sciences, Queensland University of Technology, Brisbane, Queensland, 4000, Australia; 3 Centre for Musculoskeletal Research, Griffith University, Southport, Queensland, 4222, Australia; 4 Department of Anatomy and Developmental Biology, Monash University, Clayton, Victoria, 3800, Australia; 5 Australian Regenerative Medicine Institute, Monash University, Clayton, Victoria, 3800, Australia; Team 'Evo-Devo of Vertebrate Dentition', FRANCE

## Abstract

The origin of terrestrial tetrapods was a key event in vertebrate evolution, yet how and when it occurred remains obscure, due to scarce fossil evidence. Here, we show that the study of palaeopathologies, such as broken and healed bones, can help elucidate poorly understood behavioural transitions such as this. Using high-resolution finite element analysis, we demonstrate that the oldest known broken tetrapod bone, a radius of the primitive stem tetrapod *Ossinodus pueri* from the mid-Viséan (333 million years ago) of Australia, fractured under a high-force, impact-type loading scenario. The nature of the fracture suggests that it most plausibly occurred during a fall on land. Augmenting this are new osteological observations, including a preferred directionality to the trabecular architecture of cancellous bone. Together, these results suggest that *Ossinodus*, one of the first large (>2m length) tetrapods, spent a significant proportion of its life on land. Our findings have important implications for understanding the temporal, biogeographical and physiological contexts under which terrestriality in vertebrates evolved. They push the date for the origin of terrestrial tetrapods further back into the Carboniferous by at least two million years. Moreover, they raise the possibility that terrestriality in vertebrates first evolved in large tetrapods in Gondwana rather than in small European forms, warranting a re-evaluation of this important evolutionary event.

## Introduction

A pivotal phase in vertebrate history was the evolution of tetrapods from sarcopterygian fish, and their subsequent colonization of land [1]. In addition to radical anatomical changes [2–8], it encompassed a profound shift in functional morphology and behaviour, due to the increased effect of gravity out of the support of water. However, exactly how and when tetrapods adapted to the requirements of a terrestrial lifestyle remains elusive. Based on current understanding of Late Devonian tetrapods, the advent of vertebrate terrestrialization is inferred to have taken place during the Early Carboniferous [1], yet tetrapod fossils are extremely scarce from the first 30 million years of this period, approximately 359–329 million years ago (mya). The oldest known incontrovertibly terrestrial tetrapods are small animals from the Late Viséan of Scotland [9,10], approximately 327–331 mya, but a global scarcity of tetrapod fossils from earlier in the Carboniferous hinders an assessment of how and when the colonization of land began [1,11,12].

**Fig 1:**
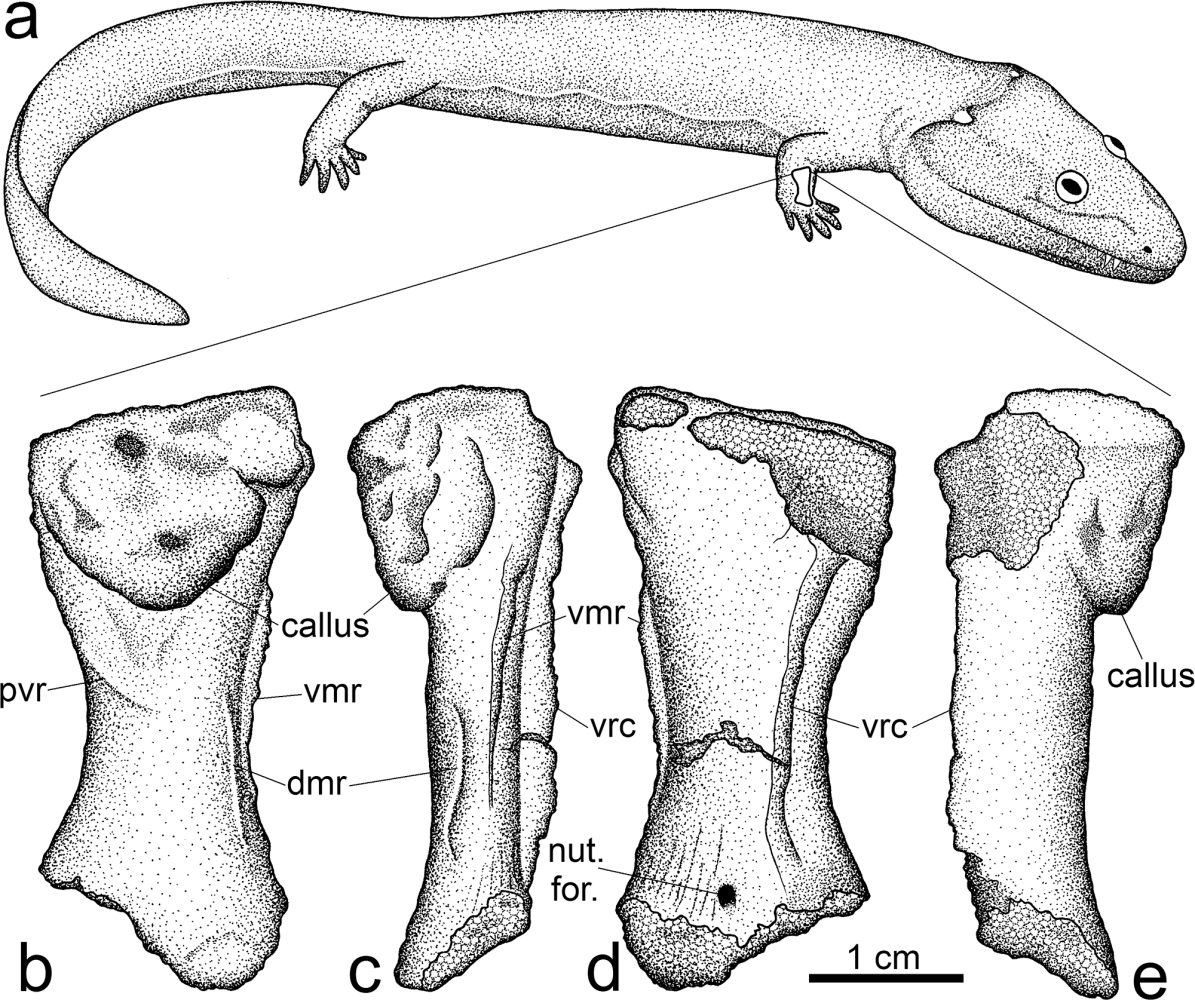
Morphology of *Ossinodus* and its fractured right radius, QMF 37451. (a) Reconstructed life appearance of *Ossinodus*, based on numerous cranial and postcranial remains; the individual to which QMF 37451 belonged is estimated to have been about 1–1.5 m long. (b–e) External morphology of radius in lateral (b), anterior (c), medial (d) and posterior (e) views; proximal (elbow) articulation to top of page. The large callus on the proximolateral surface resulted from healing of the fracture. Abbreviations: dmr, dorsomesial ridge; pvr, proximoventral ridge; vmr, ventromesial ridge; vrc, ventral radial crest; nut. for., nutrient foramen.

Deriving from the mid-Viséan (333 mya) of Queensland, Australia [13,14], the primitive stem tetrapod *Ossinodus pueri* (Fig 1a) is of critical importance to interpreting the evolution of terrestrial tetrapods. Along with the Late Devonian *Tulerpeton* [15], *Ossinodus* is remarkable among early stem tetrapods in the high degree of ossification in its appendicular elements, comparable to that of many early amniotes [14,16]. All *Ossinodus* bones have been recovered from a single site preserving multiple individuals of varying size [14], and all stem tetrapod bones from this site are regarded as belonging to *O*. *pueri*, based on a similar pattern of ornamentation on the recovered dermal cranial bones [14,16]. Among the numerous cranial and postcranial elements recovered for *Ossinodus* is a fractured right radius, which is the oldest known tetrapod palaeopathology [17] (Fig 1b–1e). The presence of a callus shows that healing had commenced, but given that the fracture can still be observed in X-radiographs of the fossil [17], this indicates that the radius had only partially healed when the individual died. Having fractured when the individual was alive, it can therefore provide insight into stem tetrapod lifestyle and behavior [18].

The fossil’s excellent preservation presents the unique opportunity to analyze the fracture’s three-dimensional geometry via high-resolution computed tomographic (CT) scanning. The CT scanning also permits the assessment of the internal microstructure of the fossil, such as that of cancellous bone. Additionally, we employed high-resolution, three-dimensional finite element analysis (FEA), an approach that has been used to accurately simulate structural mechanics in both living and extinct taxa [19,20], to determine the conditions under which fracturing occurred. This provides direct evidence of lifestyle in a stem tetrapod, which has important implications for understanding the physiological, temporal and biogeographical context under which terrestriality in vertebrates evolved.

## Materials and Methods

No permits were required for the described study, which complied with all relevant regulations.

### Data acquisition

The fractured right radius of *Ossinodus*, Queensland Museum Fossil specimen (QMF) 37451, was scanned using a Bioscan NanoPET/CT scanner. The settings used are as follows: helical scan method, 1100 ms exposure time, 65 kV peak voltage, nominal isotropic voxel resolution of 65 μm. In order to achieve adequate spatial resolution (i.e., a resolution of 65 μm), the fossil was scanned in three parts along its long axis, and the resulting scan series rejoined in Avizo 7.0 (Visualization Sciences Group, USA).

### Analysis of cancellous bone architecture

The trabecular architecture of cancellous bone was most clearly resolved in the CT scans of the proximal end of the radius. This region was quantitatively analyzed for architectural anisotropy using the mean intercept length method as implemented in the software Quant3D [21]. The region of bone analyzed was centrally located in the proximal end and occupied a volume of dimensions 5.92 × 6.50 × 4.23 mm. The entire volume was analyzed using the following settings in Quant3D: adaptive iterative thresholding, 2000 points and 2049 uniform orientations with random rotation and dense vectors. The resulting mean intercept length tensor was used to produce a fabric ellipsoid with principal axes in Rhinoceros 4.0 (McNeel, USA).

### Image processing and analysis

**Fig 2:**
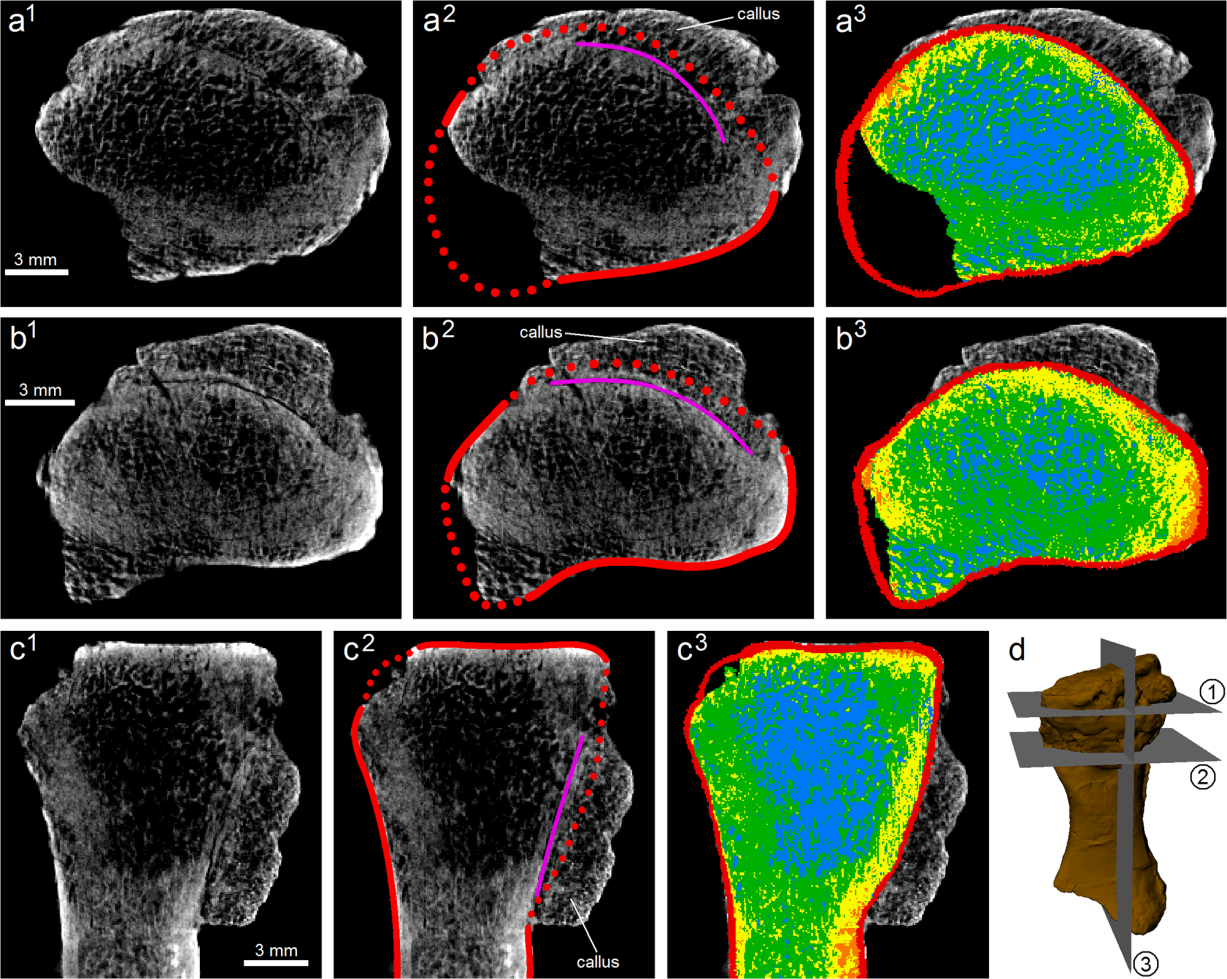
Digital removal of the callus, reconstruction of the original bone morphology, and CT image segmentation. This process is shown with three (a–c) different sections through the fossil. (d) Location and orientation of sections (a)–(c) relative to the bone. ^1^, The original scan images. ^2^, Interpretation of the scan images. Red line is preserved cortical margin, dotted red line is reconstructed cortical margin, and purple line is the fracture. The axially concentric pattern of density change (brighter pixels indicating higher density) within the bone allowed estimation of the position of the original cortical margin, based on the amount of the concentric pattern missing. Note the finer, grainer microstructure of the callus. ^3^, Scan image segmented into cortical bone (red) and four different densities of cancellous bone (orange, yellow, green, and blue, in decreasing order of density).

The CT scans were visualized, segmented, three-dimensionally modelled and analyzed in Mimics 14.0 (Materialize NV, Belgium), Rhinoceros and the open source software ImageJ 1.47 (http://rsb.info.nih.gov/ij/). The pathological fracture was identified in the scans as a sharp discontinuity with a long, linear geometry, manifest as an area much darker (i.e., lower greyscale values) than the surrounding bone (Fig 2); its orientation and extent was subsequently mapped out manually. It was distinguished from post-mortem, preservational fractures on the basis that preservational fractures extended to one or more areas at the bone surface, and that they were typically wider (several pixels across) than the pathologic fracture (generally two pixels across). Identifying the orientation of the fracture, and the sense of fracture displacement, allows for the loading conditions under which fracturing occurred to be reconstructed.

**Fig 3:**
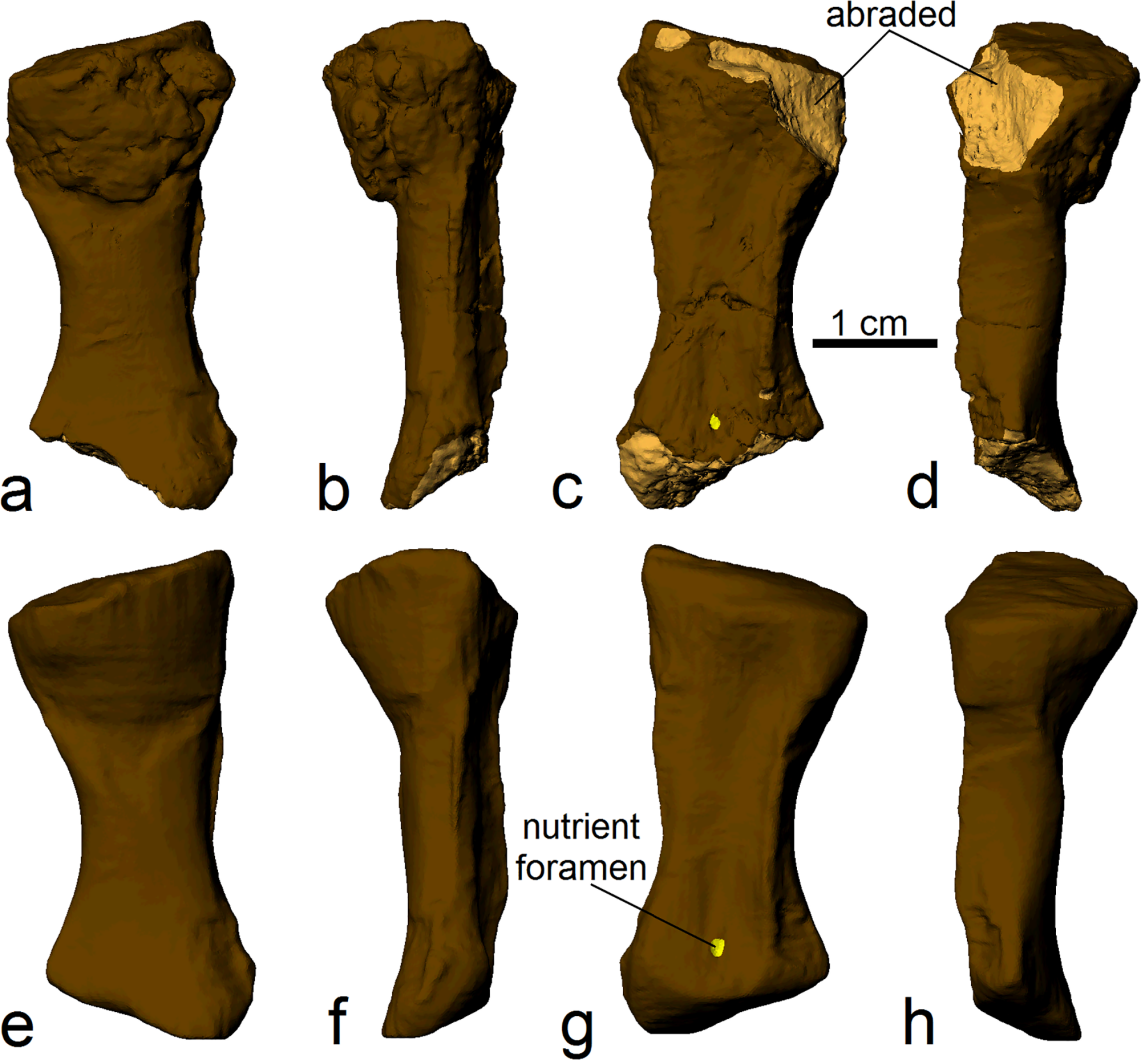
Removal of the callus and reconstruction of the pre-fractured morphology of the radius of *Ossinodus*. (a)–(d) Three-dimensional geometry of the fossil, with abraded parts highlighted proximally and distally. (e)–(h) Reconstructed three-dimensional geometry of the original bone, as it is would have appeared prior to fracturing. Views correspond to those in Fig 1b–1e.

In order for a finite element model of the radius of *Ossinodus* to be produced, the bone’s morphology prior to fracturing had to be reconstructed. This required that the following features of the fossil be digitally ‘corrected’ in the CT scans, using Mimics 14.0 (Figs 2 and 3):

1. The healing callus was removed, identified on the basis of its internal microstructure. The microstructure of the callus was much finer and grainier than that of the normal, nonpathologic bone; it also showed little trace of trabeculae. Removing the callus, however, also removes what was once original bone. Therefore, once it was removed, the original external outline of the bone had to be restored in that area; this was primarily achieved by using the concentric pattern of density change within the bone as a guide.
2. The proximal and distal ends of QMF 37451 are abraded to different degrees (Fig 3). These were restored to achieve a complete bone, partly via comparison to the morphologies of the radii of other, phylogenetically close, Early Carboniferous tetrapods [22,23]. The nature of cancellous bone within the missing parts could not be reconstructed, because there are no other known fossil radii of *Ossinodus* to obtain the information for those missing parts.
3. Preservational (taphonomic) fractures were removed by ‘filling in’ via extrapolation from adjacent regions of bone, using the appropriate density of bone (see below).

To enable the development of a heterogenous finite element model, the reconstructed morphology of the pre-fractured radius was segmented into both cortical and cancellous bone components (Fig 2). Cortical bone was segmented using a combination of thresholding tools and manual segmentation. The remaining volume of the bone (cancellous bone) was segmented into four different components with different bulk densities, via thresholding of voxel greyscale values. The range of greyscale values used for separating out each component were chosen on the qualitative appearance of the CT data; the separation used was deemed to best represent the structural patterns present, ensuring that the model subsequently generated was as realistic as possible.

### Finite element model construction

**Fig 4:**
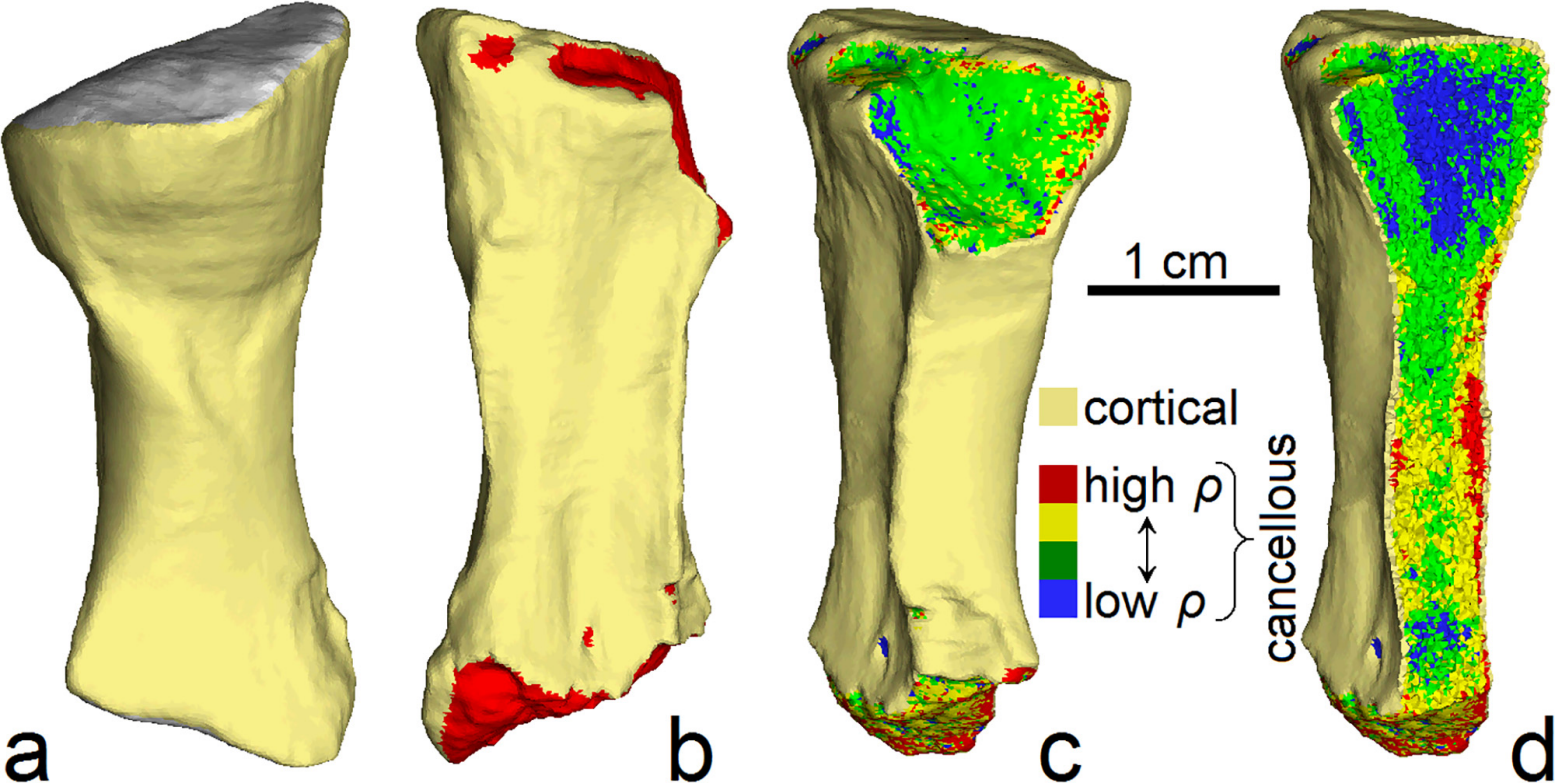
Three-dimensional finite element models of the radius of *Ossinodus*. (a) Complete homogenous model in oblique posterolateral view; extent of proximal articulation surface shown in grey. This was the model used to assess fracture mechanics in the radius. (b) Incomplete homogenous model in anteromedial view; red shows exposed (homogenous) cancellous bone. (c) Incomplete heterogenous model in posteromedial view. (d) Incomplete heterogenous model in posteromedial view, sectioned in the mediolateral plane to illustrate variation in material properties within the volume of cancellous bone.

In order to determine how and with what magnitude force the radius of *Ossinodus* would have fractured, the CT scan data was used to develop a high-resolution, three-dimensional, finite element model of the bone’s pre-fractured morphology in Strand7 2.4.4 (Strand7 Pty Ltd, Australia). The fossil’s preservation allowed for cortical bone and different components of the cancellous bone to be modelled separately based on density differences, thus enabling patterns of variation in material properties in the original, living bone to be accounted for. However, since a small part of the fossil was missing, knowledge of structural and material heterogeneity within the cancellous bone is incomplete. Thus, in the final model used, all cancellous bone elements were assigned a single set of material properties, distinct from those assigned to the cortical bone elements; each component (cortical and cancellous) was modelled as a homogenous material. This model is referred to herein as the ‘complete homogenous model’ (Fig 4a, Table 1).

**Table 1:** Material properties assigned to the elements in the three finite element models.

Material	Density, *ρ* (g/cm^3^)	Young’s Modulus, *E* (MPa)*	Poisson’s Ratio, *ν*
Cortical bone	2.06	20,000	0.4
Cancellous bone density 1†	1.6	17,500	0.3
Cancellous bone density 2†	1.4	15,000	0.3
Cancellous bone density 3†	1.2	12,000	0.3
Cancellous bone density 4†	1.1	11,000	0.3
Homogenous cancellous bone‡	1.3	13,500	0.3
Articular surfaces	1.1	10,000	0

All elements were low-order tetrahedral bricks except for the network of tessellated beams representing the articular surfaces. Values for cortical bone from [24–27].

* Values rounded to the nearest 0.5 GPa, to allow for uncertainty

^†^ Only present in the incomplete heterogenous model

^‡^ Only present in the incomplete and complete homogenous models

To assess the possible effects of modelling the cancellous bone as a single homogenous volume, two further finite element models of the preserved part of the fossil were produced for comparative analysis (Fig 4), both without any of the reconstructed parts of the proximal and distal ends of the bone. The purpose of these two models was to assess whether the representation of a spatially and compositionally heterogenous material as a single, homogenous volume (as in the complete homogenous model) would introduce significant error into the analysis of fracture mechanics. Thus, in one model, the preserved cancellous bone was represented by a single, homogenous material (with material properties distinct from those assigned to the cortical bone elements); this is herein referred to as the ‘incomplete homogenous model’ (Fig 4b, Table 1). In the other, the preserved cancellous bone was represented by four separate densities and material properties (corresponding to the four components produced in the initial segmentation); this is herein referred to as the ‘incomplete heterogenous model’ (Fig 4c and 4d, Table 1). Comparative FEA of these two models would elucidate any error introduced by the simplified representation of cancellous bone, since the only difference between the two was how the cancellous bone was represented.

To produce the complete homogenous finite element model, a rendered surface mesh of the reconstructed (pre-fracture) radius was produced in Mimics 14.0. This was then exported to Avizo 7.0, where a solid mesh of 1,189,119 low-order tetrahedral brick elements was generated. The solid mesh was re-imported into Mimics 14.0, where the segments of cortical and (homogenous) cancellous bone were used to assign different material properties to the constituent brick elements of the mesh, via the ‘mask intersection’ method built into Mimics 14.0. Finally, this was then imported into Strand7 2.4.4. A similar procedure was employed in generating the incomplete heterogenous and incomplete homogenous finite element models; both consisted of 1,080,638 low-order tetrahedral brick elements.

In lieu of a more complete understanding of the variation in bone material property anisotropy in extant tetrapods, all elements were modelled as isotropic materials, that is, they respond the same way to loading regardless of the direction the load comes from.

Cortical bone elements in all three models were assigned the same set of material properties (Table 1). Values for physical density (*ρ*), Young’s modulus (*E*) and Poisson’s ratio (*ν*) were drawn from many studies of the main limb bones of a wide variety of animals, mostly mammals and birds [24–27]. The values used here are approximately the modal values reported for each property, and they represent a conservative first estimate.

To accurately model the cancellous bone, a more involved approach was required. First, the patterns of variation in physical density in the original, living bone had to be determined. In living bone, the greyscale (Hounsfield) values in CT scans can be used to directly determine approximate physical density; however, in this case, the calculated physical densities will be that of the fossil, not the original, living bone. Nevertheless, the patterns of variation in density can be reasonably assumed to be representative of the patterns of density variation in the original bone, due to the fossil’s excellent preservation. To ensure a realistic designation, the resulting total mass of the bone in the incomplete heterogenous model was calculated following density assignment. The densities used in the final model (Table 1) yielded a total mass of approximately 4.3 g, which is a reasonable estimate of the mass of the fossil specimen if it were living bone, assuming a bulk long-bone density of 1.4 g/cm^3^ [24]. For both complete and incomplete homogenous finite element models, the physical density of the cancellous bone was the volume-weighted mean of the four densities used in the heterogenous model. Once physical densities had been determined, Young’s modulus was assigned to each element in the three models, via a regression equation derived from the data presented by [28], *E* = 0.001*ρ* ^1.3266^, where *E* is in GPa and *ρ* is in kg/m^3^. Lastly, elements were assigned values of Poisson’s ratio. Experimentally determined values for *ν* in living cancellous bone are quite variable, and it is unclear as to how this variation correlates to variation in density [29]. Thus, a conservative approach was taken here, whereby all cancellous bone elements in each model were assigned a value of *ν* = 0.3 [30], pending further data.

To represent the effects of articular cartilages at the proximal and distal ends of the radius, the inferred articular surfaces (reconstructed for the distal end) were modelled as a tessellated network of stiff beam elements. This helps to reduce the incidence of point artifacts in the results of a FEA [28,30].

### Comparative FEA of incomplete heterogenous and homogenous models

To investigate the potential error introduced by modelling a compositionally and spatially heterogenous volume of cancellous bone as a single, homogenous volume, we tested a number of different loading regimes applied to the incomplete heterogenous and incomplete homogenous finite element models:

1. Uniaxial compression directed parallel to the bone’s long axis, with loads applied to the proximal end.
2. Uniaxial tension directed parallel to the bone’s long axis, with loads applied to the proximal end.
3. Uniaxial torsion about the long axis of the bone, with a positive moment applied at the proximal end.
4. Anteroposterior bending, with an anteriorly directed force applied at the posterior surface of the bone at its midshaft.
5. Mediolateral bending, with a medially directed force applied to the lateral surface of the bone at its midshaft.

The first three models were restrained in translation at a small number of nodes in the centre of the distal end; the last two were restrained in translation at a small number of nodes in the centre of both ends. Each model was analyzed as a linear static system; hence, the magnitude of applied forces in a given loading regime is unimportant, only that they are consistent between models.

### FEA of complete homogenous radius model

Based on the identified orientation and nature of displacement of the fracture (see Results and Discussion), the complete homogenous finite element model was used to identify the loading regime that caused the radius to fracture. To prevent free-body movement of the model under loading, the distal end was restrained in translation at a small number of nodes in the centre. Given that the fracture and bone presents features associated with impact-type situations (trauma), rather than those associated with bone fatigue (stress fractures), tendon avulsion, or prior pathology [17,18], all force vectors used in a given loading regime (simulating the external, fracture-causing load) were of the same magnitude and orientation. The applied loads did not incorporate any ‘background loading’ as might occur during normal use, such as that produced by muscular action. This is because strain in the area of interest (the proximal end of the bone) is typically very low during normal use [31]. Hence, strain results in the proximal end of the model will almost completely reflect the applied external loads.

To determine which loading regime was responsible for the fracture, each model was analyzed as a linear static system, and the results were assessed according to two criteria:

1. Strain field pattern: the correct loading regime would result in regions of high von Mises strain (a measure of distortion) which are aligned with the orientation of the fracture and are spatially restricted to the extent of the fracture. It has been suggested that von Mises *stress* is a good predictor of failure in bone [30], although in a linearly elastic model it will show the same pattern as its strain equivalent.
2. Stress field pattern: the desired loading regime will produce a stress field in which the orientation of maximum (*σ*_11_), minimum (*σ*_33_), and intermediate (*σ*_22_) principal stresses in the region of the fracture are consistent with shear fracturing along the orientation of the fracture plane and in the correct direction of displacement [32]. In particular, *σ*_11_ is at a high angle to the fracture plane, *σ*_33_ is at a low angle to the fracture plane, and *σ*_22_ is parallel to the fracture plane and perpendicular to the direction of slip.

The identification of the loading regime that satisfied these criteria proceeded in an iterative ‘trial and error’ fashion. Recognizing that the mode of fracture (see Results and Discussion) required a force applied somewhere on the proximolateral end of the bone and with a component directed distally [32–34], this was used as an initial starting point for force vector orientation and area of application in the FEA. The results from this loading regime were used to guide the set-up of the next loading regime (i.e., alter the orientation of the force vectors, or its area of application, or both), and so on until a solution was achieved.

## Results and Discussion

### Fracture and callus

**Fig 5:**
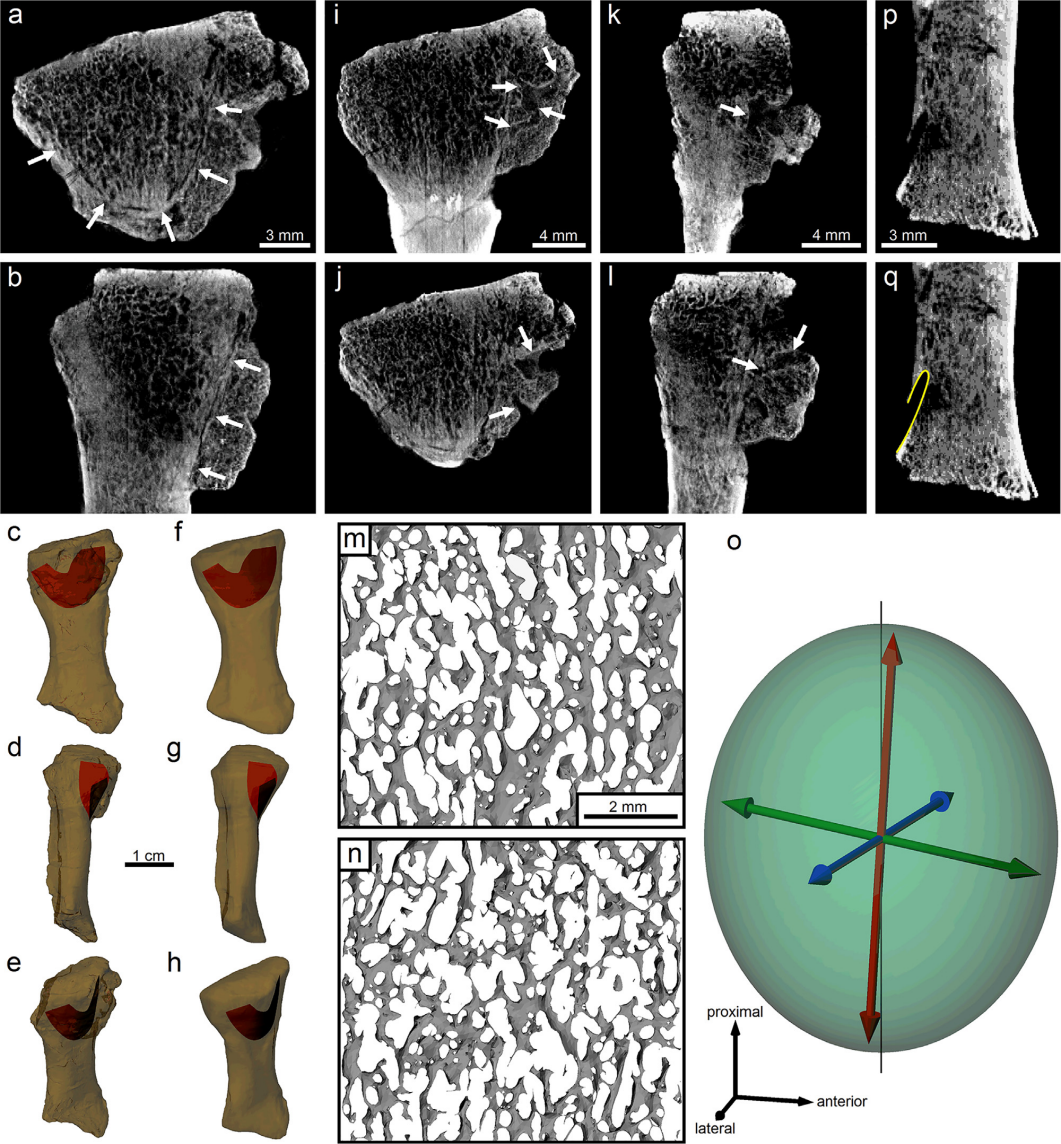
Internal microstructural features in the radius of *Ossinodus*, QMF 37451. (a), (b) CT images showing fracture geometry in mediolateral (a) and anteroposterior (b) views; the arrows delimit the shape and extent of the fracture, and are on the callus side of the fracture. (c)–(h) Three dimensional geometry of the fracture (red) superimposed on the original (c–e) and reconstructed (f–h) morphology of the radius; (c), (f) are in lateral view; (d), (g) are in posterior view; (e), (h) are in oblique posterolateral view. (i)–(l) Lesions and subsurface canals in the callus as they appear in CT scans, shown in both mediolateral (i, j) and anteroposterior (k, l) views; arrows delimit extent of the canals. (m), (n) Cancellous bone architecture in the proximal end of the bone, shown as smoothed renderings of volumes automatically segmented from CT scans; (m) Bone in a volume measuring 7.4 × 6.5 × 0.18 mm, with proximal to top of page, anterior to right; (n), bone in a volume measuring 7.4 × 6.5 × 0.23 mm, with proximal to top of page, anterior to left; these regions correspond to that which was quantitatively analyzed for architectural alignment. (o) Fabric ellipsoid (and principal axes) for cancellous bone in the proximal end of the radius; note the almost perfect alignment of the primary fabric axis (red vector) with the proximodistal axis of the bone (thin black line). (p), (q) Morphology of a single nutrient foramen piercing the distal medial end of the bone; (p) shows CT image in anteroposterior view, through the middle of the foramen; (q) shows the same image, with the extent of the foramen (diameter 0.7 mm) outlined in yellow; note the low angle of entry into the bone surface.

The CT scans reveal a single fracture plane in the proximal end of the radius (Fig 5a–5h). The fracture lies just beneath the endosteal surface, and is parabolic in mediolateral view and almost concentric with the margins of the bone in transverse section. The healing process has produced a callus, which is perforated by six sizeable lesions (pits) that occasionally form subsurface canals (Fig 5i–5l), indicating post-traumatic infection of the bone, with pus formation (suppurative osteomyelitis) [35]. The callus is demarcated distally by a pronounced groove, where the fracture nears the bone surface (Fig 5b and 5d).

### Osteological microstructure

The architecture of cancellous bone in the proximal end of the radius has a preferred trabecular orientation (Fig 5m and 5n), with a degree of anisotropy (the relative magnitudes of primary and tertiary material eigenvectors [21]) of 1.27. Moreover, the primary axis of the fabric ellipsoid is almost perfectly aligned with the bone’s long axis (Fig 5o): the axis of primary trabecular alignment is essentially parallel to the long axis of the bone. This suggests that the radius underwent remodelling throughout life as a response to frequent, axially directed loads [25,36–39]. This would occur if the animal spent considerable time on land with the limbs in a sprawling position, and the forearm held approximately vertically to support the animal’s weight. This interpretation receives further support from the morphology of a single nutrient foramen which pierces the distal medial surface of the radius (Fig 5p and 5q). The low-angle of entry (~20°) into the bone would theoretically reduce potentially dangerous stress concentrations produced under axial loading [25], thus serving as an additional adaptation to weight support.

### Heterogenous versus homogenous finite element models

**Fig 6:**
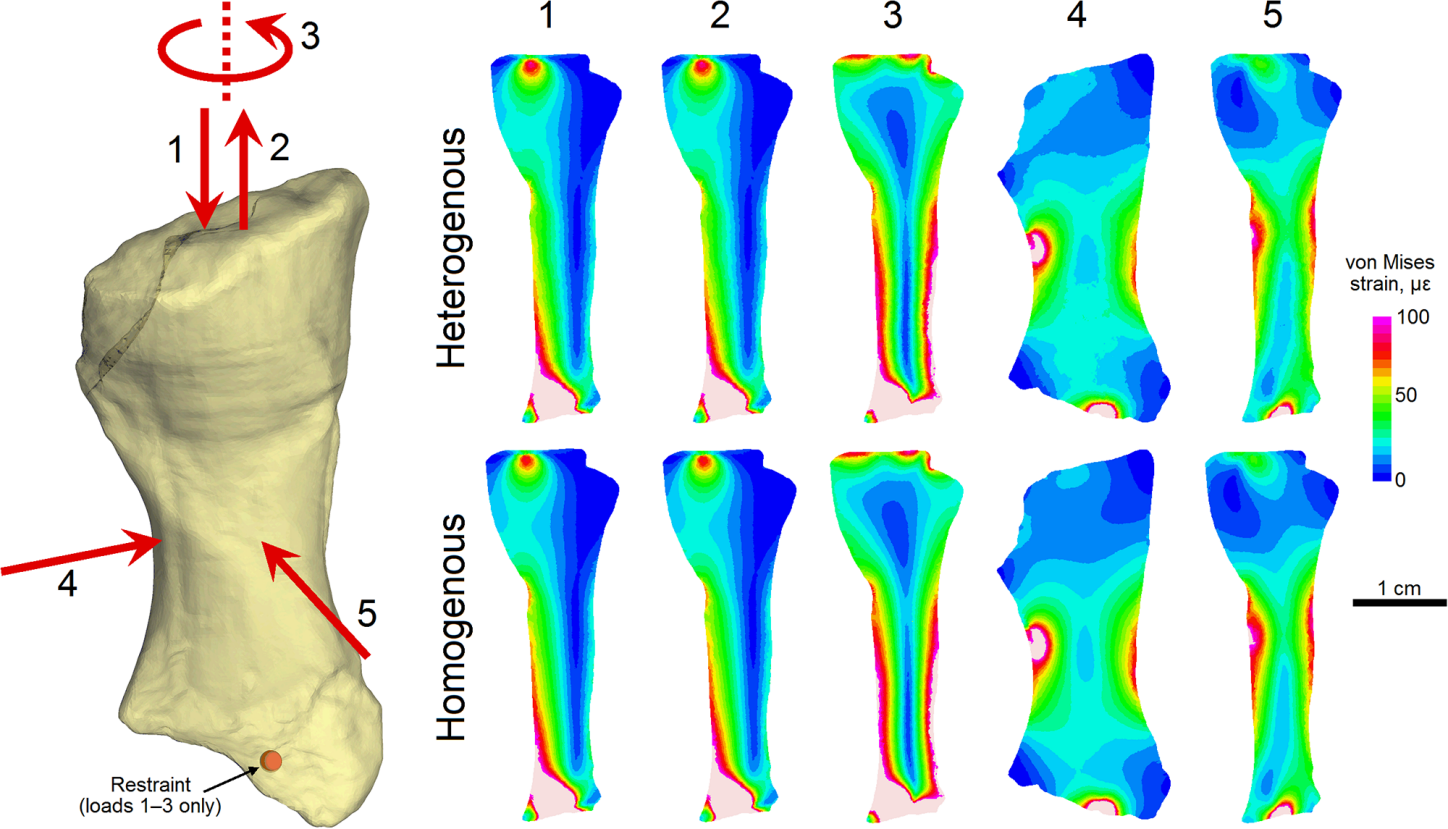
Comparative FEA of incomplete heterogenous and incomplete homogenous finite element models. Five different loading regimes were applied to both models (left) with resulting von Mises strain patterns compared via cross sectional plots (right). 1 = uniaxial compression, 2 = uniaxial tension, 3 = uniaxial torsion, 4 = anteroposterior midshaft bending, 5 = mediolateral midshaft bending. All plots are mediolateral cross sections through the middle of the bone, except for loading regime 4, which is shown as an anteroposterior cross section through the middle of the bone; values reported as microstrain (με = 1 ×10^-6^); light pink indicates regions of strain higher than 100 με. Note the great similarity between the heterogenous and homogenous model results under each loading regime.

The comparative FEA between the incomplete heterogenous and incomplete homogenous finite element models revealed no significant difference in their biomechanical performance under each loading regime tested (Fig 6), consistent with the results of previous work [40]. Both models predicted very similar stress and strain field patterns; furthermore, comparison of mean von Mises brick strain [28] between the two models for each loading regime showed very small differences in model results (Table 2), with a maximum difference of ~1.5%. Importantly, this means that a complete model with a homogenous volume of cancellous bone captures the biomechanical behaviour of the bone with sufficient accuracy to address the question of fracture mechanics in the radius of *Ossinodus*.

**Table 2:** Results of comparative FEA between heterogenous and homogenous models.

Loading regime	Heterogenous (με)	Homogenous, (με)	Difference in means relative to heterogenous (%)
Uniaxial compression	24.010 [243.416]	24.134 [232.951]	0.515
Uniaxial tension	24.010 [243.416]	24.134 [232.951]	0.515
Uniaxial torsion	42.002 [336.307]	42.649 [325.280]	1.541
Anteroposterior bending	18.195 [49.845]	18.421 [50.137]	1.243
Mediolateral bending	21.427 [26.759]	21.519 [27.810]	0.426

Outlines comparison between von Mises brick strain results (reported as mean [standard deviation]) for both the incomplete heterogenous and incomplete homogenous models under each of the five test loading regimes.

### Evaluation of fracture scenario

**Fig 7:**
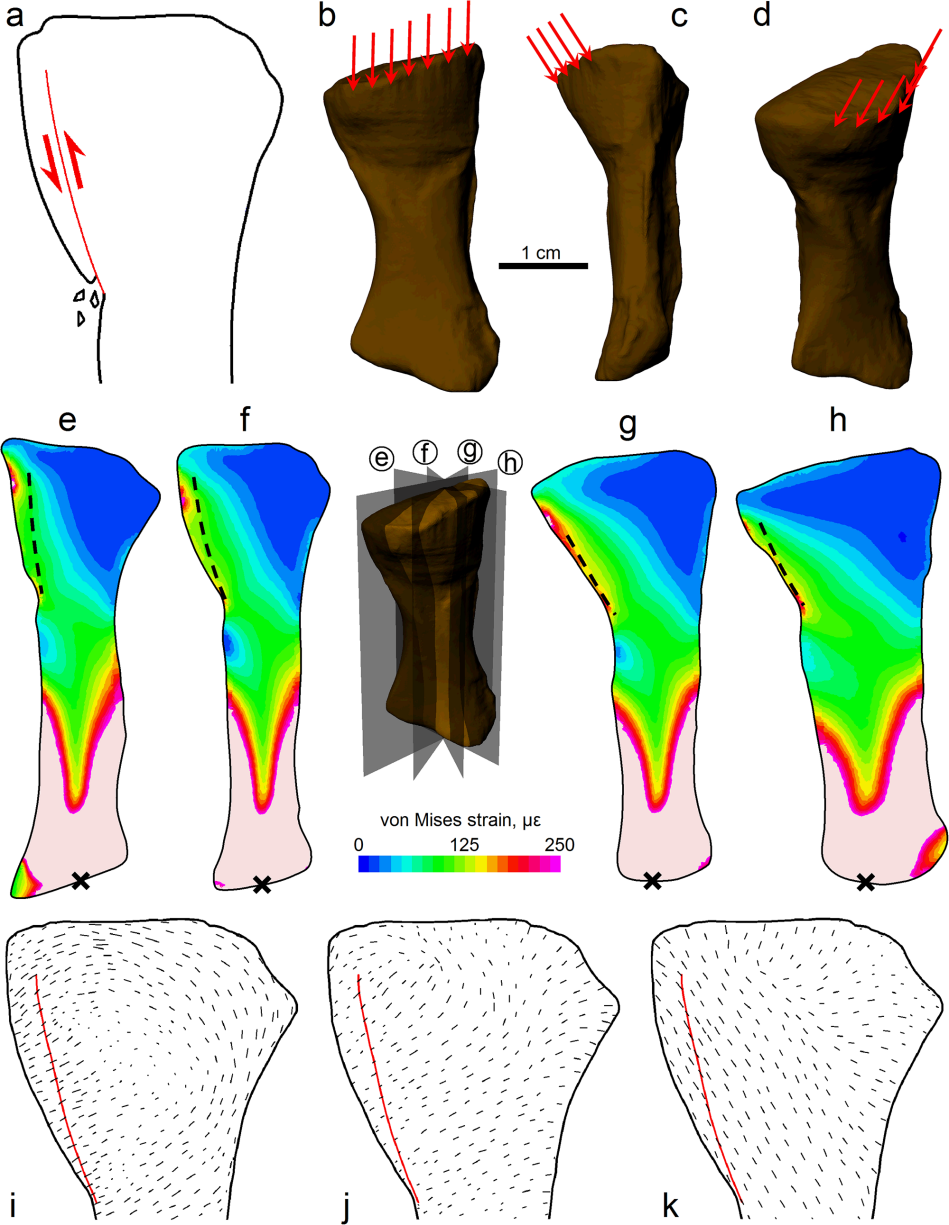
Fracture mechanics and comparison of FEA results with fracture geometry. (a) Fracture via distal displacement of an external fragment (or fragments). (b)–(d) Relative orientation and approximate area of application of the fracturing force, shown in lateral (b), anterior (c) and oblique posterolateral (d) views. (e)–(h) Cross sections of the radius on four different planes through 90° with von Mises strain patterns resulting from the loading of (b)–(d); fracture geometry shown as dashed line, distal restraint used in the FEA shown as a cross; inset shows orientation of each cross sectional plane. Strain values reported as microstrain; light pink indicates strains higher than 250 με. High strains in the distal end are artifacts resulting from the necessity to properly restrain the model; placing restraints as far away as possible from the region of interest (the proximal end) ensured that minimal effect was made to the proximal radius, including the fracture zone. (i)–(k) Cross sections of the radius with maximum (i), intermediate (j) and minimum (k) principal stress field patterns resulting from the loading of (b)–(d); fracture geometry shown as red line; plane of cross-section corresponds approximately to that in (f).

The orientation of the fracture and the nature of the surrounding bone allows for the sense of displacement along the fracture to be determined. Given that the fracture nears the bone surface at a distinct groove in the fossil (Fig 5b and 5d), this indicates distal displacement of an external fragment (or fragments) of bone, which subsequently healed out of position (Fig 7a).

In the FEA of the complete homogenous model, a total of nineteen different loading regimes were tested before the predicted stress and strain results were deemed to satisfy the two criteria of strain and stress field patterns. The loading regime which would have resulted in fracture was found to be a force applied to almost the entire width of the proximal-most lateral surface of the bone; this force was essentially axially directed, towards the distal end (Fig 7b–7d). This loading regime produced the expected patterns of localized high von Mises strain and a principal stress field consistent with the hypothesized mode of displacement (Fig 7e–7k).

Since the model was analyzed as a linear static system, the absolute magnitude of applied forces in the simulation was unimportant; nevertheless, the simulation does facilitate estimation of the magnitude required for fracture. In the simulation, the applied force which satisfied the above criteria had a magnitude of 56.1 Newtons (N), and peak von Mises strain in the fracture zone in this solution was approximately 300 με (Fig 7e–7h). The yield strain of bone may be assumed to be 6,000 με [25]. In a linear system, strain is directly proportional to stress, and hence force; therefore, the actual force required for fracture is

$$\begin{array}{l} F\;\;=\;\;56.1\;\;\times \;\left(6,000\;÷\;300\right)\\ \approx 1,120\;\text{N}. \end{array}$$

Dividing by gravitational acceleration (*F* = *ma*_g_, where *a*_g_ = 9.8 m/s^2^), this is equivalent to a loading mass of *m* = 114 kg.

To express this loading mass in terms of the *Ossinodus* individual concerned requires that its body mass is known. Given that *Ossinodus* likely had body proportions similar to that of many other early tetrapods from the Late Devonian to Early Carboniferous, especially *Pederpes* (Fig 1a; see also [16,22,41]), the individual the radius belonged to is estimated as having a total (snout—tail) length of 1–1.5 m. (The range in the estimate given here is due in large part to uncertainty in the relative length of the tail; see [41].) The mass of this individual may be estimated via comparison to the Chinese giant salamander, *Andrias davidianus*, which reaches total lengths of 2 m and masses of 50 kg [42], and has body proportions similar to that of many early tetrapods. For an *Ossinodus* of length *l*_Ossinodus_ = 1–1.5 m, its mass *m*_Ossinodus_ would hence be

$$\begin{array}{l} m_{\text{Ossinodus}}\;=\;m_{\text{Andrias},\text{ max}}\;\times \;{\left(l_{\text{Ossinodus}}\;÷\;l_{\text{Andrias},\text{ max}}\right)}^{3}\\ \;=\;50\;\times \;{\left(1\;÷\;2\right)}^{3}\;\text{to }\;50\;\times \;{\left(1.5\;÷\;2\right)}^{3}\\ \;=\;6.3\;\text{ to}\;21.1\;\text{kg}. \end{array}$$

To be conservative (in terms of calculating the relative magnitude of the impact force) the mass of the *Ossinodus* individual is estimated to be 10–25 kg. Expressed in terms of the animal’s body weight, the fracturing force is therefore

$$\begin{array}{l} F\;=\;\;114\;÷\;10\;\text{ to}\;114\;÷\;25\\ \;\approx 5\;\text{ to}\;11\;\text{body weights}. \end{array}$$

Crucially, forces of this large relative magnitude are very difficult to achieve in water, because of drag and the cushioning effect water has on impacting bodies [27,43]. In contrast, these large forces could easily be experienced on land, such as falling off a log or rock, or being caught in the collapse of unstable ground.

The plausibility of a fall scenario is demonstrated here using basic Newtonian mechanics. Consider a situation in which an *Ossinodus* of mass *m* = 25 kg falls some distance *s*_h_, experiencing an impact force of *F* = 1,120 N. Important to the force experienced is the distance *s*_d_ over which the body decelerates from free-fall to a standing stop on the ground (with deceleration *a*), which may be assumed to be approximately equal to the dorsoventral depth of the animal’s body, say 0.15 m [16]. Slowing down to a final velocity of *v*_f_ = 0 m/s from free-fall, the maximum free-fall velocity reached prior to deceleration, *v*_max_, is:

$$\begin{array}{l} v_{\text{f}}^{\;2}\;=\;v_{\text{max}}^{\;2}\;+\;2as_{\text{d}}\\ v_{\text{max}}^{\;2}\;=\;\;v_{\text{f}}^{\;2}\;-\;2\;\times \;-\left(F/m\right)\;\times \;s_{\text{d}}\\ \;=\;0^{2}\;-\;2\;\times \;-1,120/25\;\times \;0.15\\ \;=\;13.44{\text{ m}}^{2}/{\text{s}}^{2},\text{ that is},\\ v_{\text{max}}\;=\;\;3.67\text{m}/\text{s}. \end{array}$$

The height of free-fall can now be calculated, assuming a standing start (*v*_i_ = 0 m/s):

$$\begin{array}{l} v_{\text{max}}^{\;2}\;=\;\;v_{\text{i}}^{\;2}\;+\;2a_{\text{g}}\left(s_{\text{h}}\;-\;s_{\text{d}}\right)\\ 13.44\;\;=\;0^{2}\;+\;2\;\times \;9.8\;\times \;\left(s_{\text{h}}\;-\;s_{\text{d}}\right)\\ s_{\text{h}}\;-\;s_{\text{d}}\;\approx \;0.7\;\text{m}\\ \text{thus }s_{\text{h}}\;\approx \;0.85\;\text{m}. \end{array}$$

That is, a 25 kg *Ossinodus* would only have to fall 85 cm to sustain the impact force of 1,120 N sufficient to fracture its radius: the large forces required for fracture could be easily achieved in a fall on land. This is the minimum distance required to fall, based on the uppermost limit of estimated body mass (25 kg). If a lighter animal was involved, the corresponding fall distance would increase; for example, if a 15 kg animal were involved (and the *s*_d_ is scaled appropriately to reflect the smaller depth of the animal’s body), it would have to fall about 110 cm. As all of the estimations and assumptions underpinning the above calculations have been made on the conservative side (e.g., the purposeful overestimation of body mass), we are confident that the force required for the radius to fracture in the manner it did was very large, and that a fall on land is entirely possible from a mechanical perspective. Consistent with a fall hypothesis, the mode of displacement along the fracture (Fig 7a) is very similar to that of proximal radius fractures in humans which result from a fall onto an outstretched arm, where the humerus impacts upon the radial head [33,34]. Since the force required for fracture in the FEA was a distributed load, this excludes the possibility that it resulted from a bite (predatory or otherwise), where an impacting tooth would produce a spatially concentrated load. We therefore conclude that the most plausible explanation for the fracture in the radius of *Ossinodus* was that the animal was living on land and sustained a fall.

## Conclusions

This study demonstrates the potential utility of a combined palaeopathological-biomechanical approach for elucidating the lifestyle of extinct organisms. In addition to the results of FEA, we have observed two anatomical features in the radius of *Ossinodus*—cancellous bone architecture with preferential trabecular alignment parallel to the long-axis of the bone, and nutrient foramina that pierce the bone at low angles of entry—that likely would have served as important adaptations to terrestrial weight support. A consilience of the three lines of evidence reported here strongly suggest that *Ossinodus* spent a significant part of its life on land, which is augmented by its exceptional degree of ossification, presaging the condition observed in later amniotes. Concluding that *Ossinodus* was at least partly adapted to a terrestrial lifestyle then, the skeletal morphology shown by *Ossinodus* may be used as a basis for interpreting the lifestyle of other stem tetrapods. For instance, comparison of the gross osteology of known elements for *Ossinodus* and *Tulerpeton* [15], as well as *Pederpes* [22], suggests that these stem tetrapods may too have been at least partly terrestrially adapted, although this remains to be further tested.

*Ossinodus* is the oldest biomechanically demonstrable, terrestrially adapted tetrapod, being at least two million years older than *Casineria kiddi* [10], and at least five million years older than the East Kirkton tetrapod assemblage [9]. These small (generally less than 40 cm long), Scottish tetrapods have previously been widely regarded as the oldest known terrestriality adapted vertebrates [1,9,10]. Moreover, the large size of certain recovered fossil bones of *Ossinodus*, such as the skull table, cleithrum and interclavicle [14], when scaled according to its likely body proportions [41] suggests that some *Ossinodus* individuals may have reached over 2 m in length. (That is, the radius which formed the basis for the current study quite probably belonged to an individual which was not fully grown.) The finding that large, terrestrially adapted tetrapods were already present in Gondwana by the mid-Viséan raises the possibility that the first terrestrial vertebrates were not small European forms, but large animals from Gondwana. This in turn necessitates a revision of our current understanding of the physiological and biogeographical context in which terrestriality in vertebrates evolved.
